# Untreated substance use disorder affects glycemic control: Results in patients with type 2 diabetes served within a network of community-based healthcare centers in Florida

**DOI:** 10.3389/fpubh.2023.1122455

**Published:** 2023-03-16

**Authors:** Viviana E. Horigian, Renae D. Schmidt, Rui Duan, Daniel Parras, Katherine Chung-Bridges, Jacob N. Batycki, Kevin Espinoza, Peyman Taghioff, Sophia Gonzalez, Carly Davis, Daniel J. Feaster

**Affiliations:** ^1^Department of Public Health Sciences, University of Miami Miller School of Medicine, Miami, FL, United States; ^2^Health Choice Network, Miami, FL, United States; ^3^Ross University School of Medicine, Miramar, FL, United States; ^4^UHealth IT, University of Miami, Miami, FL, United States

**Keywords:** glycemic control, type 2 diabetes, substance use disorder, substance use treatment, Federally Qualified Health Centers

## Abstract

**Introduction:**

Patients with diabetes and comorbid substance use disorders (SUD) experience poor diabetes management, increased medical complications and mortality. However, research has documented that patients engaged in substance abuse treatment have better management of their comorbid conditions. The current study examines diabetes management among patients with type 2 diabetes, with and without comorbid SUD, receiving care at Florida-based Federally Qualified Health Centers (FQHC) of Health Choice Network (HCN).

**Methods:**

A retrospective analysis was conducted using deidentified electronic health records of 37,452 patients with type 2 diabetes who received care at a HCN site in Florida between 2016 and 2019. A longitudinal logistic regression analysis examined the impact of SUD diagnosis on achievement of diabetes management [HbA1c < 7.0% (53 mmol/mol)] over time. A secondary analysis evaluated, within those with an SUD diagnosis, the likelihood of HbA1c control between those with and without SUD treatment.

**Results:**

The longitudinal assessment of the relationship between SUD status and HbA1c control revealed that those with SUD (N = 6,878, 18.4%) were less likely to control HbA1c over time (OR = 0.56; 95% CI = 0.49–0.63). Among those with SUD, patients engaged in SUD treatment were more likely to control HbA1c (OR = 5.91; 95% CI = 5.05–6.91).

**Discussion:**

Findings highlight that untreated SUD could adversely affect diabetes control and sheds light on the opportunity to enhance care delivery for patients with diabetes and co-occurring SUD.

## Introduction

The number of individuals with diabetes continues to rise in the US, with ~37 million Americans having either diagnosed or undiagnosed diabetes. An estimated 90–95% of these cases are type 2 diabetes (1). The increasing prevalence of type 2 diabetes in the US is responsible for premature mortality, lost productivity, and elevated healthcare costs (2). To enhance the quality of healthcare delivery, optimize diabetes management, and improve outcomes, several sets of indicators have been developed to assess diabetes care quality (3–5). Despite these efforts, challenges persist in closing the diabetes management and care gap. Disparities in diabetes care and management are prominent in individuals with lower socioeconomic status, that are of racial or ethnic minority groups. These inequities are driven by challenges in health care access, access to medications, neighborhood resources and the social determinants of health (6, 7). To address unmet health care needs, community based Federally Qualified Health Centers (FQHCs) provide primary and preventive care to the underserved and uninsured, regardless of their ability to pay (8). Patients seeking treatment in any of the 1,375 FQHC facilities across the US constitute many of the nation's working poor, unemployed and undocumented (9). FQHC patients in Florida represent a particularly vulnerable group, as Florida is one of 12 states which has not expanded Medicaid coverage (10). This has left under- and uninsured individuals in Florida with few healthcare options, making FQHCs the safety net for high-risk, low socioecomomic status individuals who have diabetes.

The diabetes management gap is also notable in patients with diabetes and other comorbid conditions. Research shows that most people with type 2 diabetes have a comorbid condition (11, 12) that can complicate achieving desired glycemic control. Specifically, patients with comorbid diabetes and substance use disorders (SUD) experience poor diabetes management that increases the risk of lower-limb amputations, preventable diabetes-related hospitalizations, medical complications, and mortality (13–17). These unfortunate outcomes result from lack of adherence to medication treatment (18), laboratory testing (19), and other self-management behaviors such as diet (20). Despite these documented outcomes, screening for SUD in primary care continues to be a barrier driven by provider and patient stigma (21). To address SUD and combat associated adverse outcomes, the effectiveness of substance abuse treatment has been extensively demonstrated (22–30). In fact, research has documented that patients engaged in substance abuse treatment have better management of their comorbid conditions, and better adherence to medical treatments (31). However, integration of SUD treatment into mainstream of care and adoption of evidence-based interventions such as medications for Opioid use disorder is still lagging (32–34). Approximately one in seven Americans report experiencing an SUD. Given the high prevalence of type 2 diabetes and SUD, the intersection of these two conditions is an important comorbidity to understand and manage (35).

While previous studies have documented adverse outcomes in patients with type 2 diabetes and SUD with the use of electronic health care records, these studies have been conducted in large healthcare systems and academic settings (13, 16, 18, 20, 36). To our knowledge no studies have previously examined glycemic control in the type 2 diabetes and SUD population served in FQHCs, with the use electronic health records (EHR). Previous studies within this population have not examined the role of substance abuse treatment engagement in outcomes. The current study examines diabetes management among patients with type 2 diabetes, with and without comorbid SUD, receiving care at Florida centers of Health Choice Network, Inc. (HCN), a network of FQHCs. We hypothesized that patients with comorbid type 2 diabetes and SUD would be more likely to demonstrate worse diabetes management than those without an SUD; and that among a subsample of patients with an SUD, those who are not engaged in treatment for their SUD would be more likely to demonstrate worse diabetes management than those who are engaged in treatment.

## Materials and methods

### Data sources

A retrospective analysis was conducted using a limited dataset of patients with type 2 diabetes, 18–75 years old, who received care within the HCN network of FQHCs in Florida between January 1, 2016 and December 31, 2019. Data were housed and analyzed within a secure server at the University of Miami Clinical and Translational Sciences Institute. The study was approved by the University of Miami Institutional Review Board on July 22, 2022.

### Study population

The study assessed demographic information recorded in EHR, including patient age at the beginning of data collection, gender, and self-identified race and ethnicity. Patient diagnoses were determined using a standard clinical diagnostic approach. The diagnostic status for type 2 diabetes was defined using relevant International Classification of Disease Ninth Edition (ICD-9) and Tenth Edition (ICD-10) diagnosis codes. Type 2 diabetes diagnosis, based on these ICD-9 and/or ICD-10 codes, was determined over a baseline period of two years, including all patients with a diagnosis between January 1, 2016 and December 31, 2017. Specific SUD status (alcohol, chemical substances, or tobacco) was defined by (1) ICD-9 and ICD-10 codes, (2) Current Procedural Terminology (CPT) codes related to SUD-specific treatment, and/or (3) key medication terms for SUD-specific medications. Similar to type 2 diabetes, SUD status was determined during the 2-year baseline (2016–2017), and only patients without any SUD diagnosis during the entire study period from 2016 to 2019 were counted in the non-SUD group. Patients with comorbid diabetes and SUD had diagnostic codes for both Type 2 diabetes and SUD as described above. A table of codes and key words are included in Supplementary Table 1.

### Engagement in treatment

Patient engagement in diabetes treatment was characterized by pharmacological treatment, using medication key terms to identify the percentage of days covered by any type 2 diabetes medication prescription during the measurement period. A table of key words are included in Supplementary Table 2.

Patient engagement in SUD treatment was characterized by both behavioral treatment and pharmacological treatment, for those with an SUD diagnosis at baseline. Behavioral treatment was identified by relevant CPT codes, while pharmacological treatment was identified by key medication terms. Both behavioral and pharmacological treatments were specific to the type of SUD diagnosis (alcohol, chemical, or tobacco). Patients were classified as “engaged” in SUD treatment if their records indicated at least two visits (behavioral treatment) AND/OR if they had at least one SUD-specific prescription (pharmacological treatment) during the measurement period (either the 2-year baseline, 2018, or 2019). Participants who met the criteria for both behavioral and pharmacological were also categorized as “engaged” in SUD treatment. This flexible and overinclusive approach was to mimic what occurs in practice. While most patients are prescribed behavioral treatment, it is possible that some patients are only prescribed pharmacological treatment, such as the case in individuals with OUD, or are prescribed both. Engagement in the pharmacological treatment component for those with tobacco use disorder (TUD) was characterized by medication keywords for BOTH nicotine replacement AND tobacco anti-craving medications. Patients without at least two visits and without specific medication key terms were classified as “not engaged” in SUD treatment during the measurement period.

### Outcome

As a clinically important indicator of diabetes management, the American Diabetes Association (ADA) threshold of HbA1c at 7.0% (53 mmol/mol) was used to assess diabetes management. Patients were considered to have “uncontrolled” HbA1c if they had any lab value ≥7.0% during the measurement period or zero HbA1c labs reported in the given year of observation. All numeric HbA1c lab values were categorized as “controlled” [HbA1c <7.0% (53 mmol/mol)] or “uncontrolled” [HbA1c ≥ 7.0% (53 mmol/mol)]. HbA1c results coded in the EHR as free text (e.g., “missing,” “null,” “error,” “too high,” etc.) or as numerical ranges above 7.0% (53 mmol/mol) (e.g., “>14,” “14%−16%,” etc.) were coded classified as “uncontrolled”. Similarly, HbA1c results coded in free text as numerical ranges <7.0% (53 mmol/mol) were coded only categorically and classified as “controlled.”

### Analytic plan

The HCN EHR dataset contains several patient-level variables in distinct datafiles, including demographics, encounters, problem lists, procedures, medications, and laboratory data. Each datafile with the fields necessary for this analysis were merged *via* unique patient IDs.

Summary statistics were computed for each of the demographic and medical characteristics of the total sample and by SUD/non-SUD subgroups. Categorical variables were summarized using frequencies and percentages, while means and standard deviations were calculated for continuous variables.

**Figure 1:**
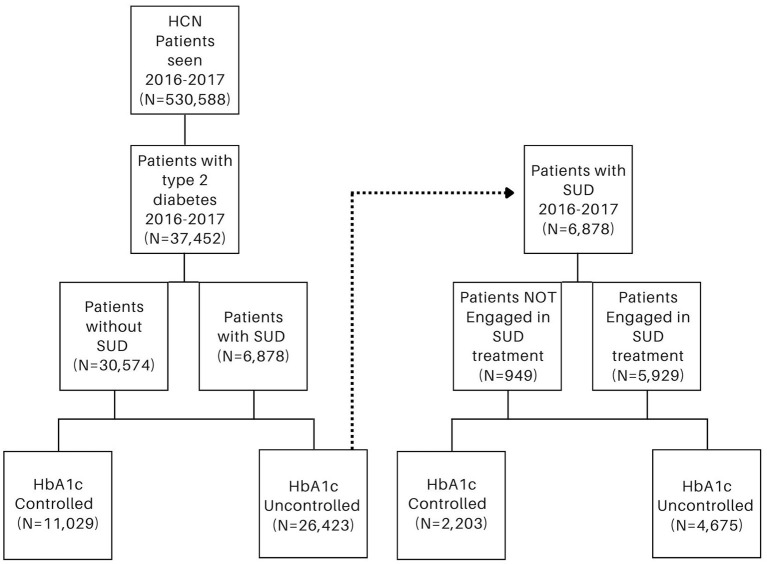
Participant sub-groups among patients with type 2 diabetes **(left)** and among patients with substance use disorder (SUD) **(right)**.

First, a generalized linear mixed model (GLMM) with a binary distribution and a logit link function was used to examine the impact of SUD diagnosis on achievement of diabetes management [HbA1c control; <7.0% (53 mmol/mol)] over time, adjusting for age, race, ethnicity, gender, and engagement in treatment for diabetes and SUD. Next, we conducted a secondary GLMM to evaluate the impact of engagement in SUD treatment (behavioral and/or pharmacological) among those with an SUD diagnosis at baseline on achievement of diabetes management over time, controlling for age, race, ethnicity, gender, and diabetes treatment (participant sub-groups shown in Figure 1). Adjusted odds ratios and 95% confidence intervals were calculated. For all analyses, two-tailed *p* < 0.05 were considered statistically significant. Both models included a random intercept for individuals to account for the repeated measures across patients and a fixed effect for clinic to account for variation among clinics. All analyses were conducted with SAS statistical software version 9.4 (37).

## Results

Among 530,588 Florida FQHC network patients, ages 18–75, served between 2016 and 2017 as identified by EHR encounters in the Problem or Procedure lists, 38,947 (7.3%) had a diagnosis of type 2 diabetes during 2016–2017 and available demographic information. Among these, 37,352 met criteria of binary SUD status of either having a baseline SUD diagnosis (6,878; 18.4%) vs. never having an SUD (30,574, 81.6%) over the study period and were included in the study. Table 1 shows the baseline characteristics of the patients overall (56.7% female, 40.5% Hispanic, 32.5% Black/African American, mean ± standard deviation age 52.9 ± 10.7 years), and among those with and without SUD.

**Table 1:** Baseline characteristics of patients with type 2 diabetes within HCN during 2016–2017.

**Characteristics**		**Total**	**Non-SUD**	**SUD**
		**(*n* = 37,452)**	**(*n* = 30,574)**	**(*n* = 6,878)**
Age		52.9 (10.7)	53.2 (10.9)	51.6 (10.0)
Gender	Male	16,339 (43.6)	12,981 (42.5)	3,358 (48.8)
	Female	21,113 (56.4)	17,593 (57.5)	3,520 (51.2)
Race/Ethnicity	Hispanic/Latinx	15,181 (40.5)	13,083 (42.8)	2,098 (30.5)
	Black/African American	12,185 (32.5)	10,108 (33.1)	2,077 (30.2)
	White	7,991 (21.3)	5,679 (18.6)	2,312 (33.6)
	Other	1,022 (2.7)	890 (2.9)	132 (1.9)
	Unknown	1,073 (2.9)	814 (2.7)	259 (3.8)
% Diabetes treatment covered days^*^		36.78 (31.9)	36.51 (31.9)	37.97 (31.6)
HbA1c controlled		11,029 (29.5)	8,826 (28.9)	2,203 (32.0)

Count and percentage [N (%)] are shown for categorical variables.

Mean and standard deviation [M (SD)] are shown for continuous variables.

^*^Percent of baseline days which a participant had any type 2 diabetes medication prescription.

Longitudinal assessment of the relationship between baseline SUD status and HbA1c control reveals that those with SUD were less likely to control HbA1c over time as compared to those without an SUD (OR = 0.56; 95% CI = 0.49–0.63). Patients who engaged in diabetes treatment were more likely to control HbA1c compared to those not engaged in treatment (OR = 5.39; 95% CI = 4.97–5.84). Likelihood of control increased with age (OR = 1.02; 95% CI = 1.02–1.03); for every year increase in age, there was an increased odds (2.0%) of having HbA1c controlled. Finally, females as compared to males (OR = 1.33; 95% CI = 1.25–1.41) and black individuals as compared to white individuals (OR = 1.21; 95% CI = 1.10–1.33) were also more likely to have controlled HbA1c (Table 2).

**Table 2:** Generalized linear mixed models (GLMM) results: substance use disorder status as a predictor of HbA1c control 2016–2019 among HCN patients with type 2 diabetes.

		**Adj. OR**	**95% CI**	***P*-value**
Age	Years	1.02	1.02, 1.03	<0.0001
Gender	Female	1.33	1.25, 1.41	<0.0001
	Male	–	–	–
Race/ethnicity	Hispanic	1.06	0.97, 1.16	0.2260
	Black	1.21	1.10, 1.33	<0.0001
	Other	0.97	0.79, 1.18	0.7440
	Unknown	0.94	0.77, 1.16	0.5826
	White	–	–	–
SUD treatment	Engaged	3.65	3.20, 4.17	<0.0001
	Not engaged	–	–	–
Diabetes treatment	%Covered days	5.39	4.97, 5.84	<0.0001
SUD status	Baseline SUD	0.56	0.49, 0.63	<0.0001
	Never SUD	–	–	–
Visit	2018	0.26	0.25, 0.27	<0.0001
	2019	0.13	0.12, 0.13	<0.0001
	Baseline	–	–	–

Among the 6,878 individuals with type 2 diabetes and a baseline SUD, 770 (11.2%) were not engaged in any SUD treatment. Among 6,108 individuals categorized as “engaged” in SUD treatment, 62 (1.0%) met criteria for only pharmacological treatment, 5,001 (81.9%) met criteria for only behavioral treatment, and 1,045 (17.1%) met criteria for both behavioral and pharmacological. Longitudinal assessment of the relationship between engagement in SUD treatment and HbA1c control reveals that those engaged in treatment were more likely to manage HbA1c over time as compared to those not engaged in SUD treatment (OR = 5.91; 95% CI = 5.05–6.91) (Table 3). Similar to results from the first model, we find among our subsample of those with an SUD, that individuals who are older (OR = 1.03; 95% CI = 1.02–1.04), females as compared to males (OR = 1.24; 95% CI = 1.08–1.43), and those engaged in treatment for their diabetes (OR = 1.85; 95% CI = 1.53–2.24), are more likely to have controlled HbA1c.

**Table 3:** Generalized linear mixed models (GLMM) results: engagement in substance use disorder treatment as a predictor of HbA1c control 2016–2019 among HCN patients with type 2 diabetes and SUD.

		**Adj. OR**	**95% CI**	***P*-value**
Age	Years	1.03	1.02, 1.04	<0.0001
Gender	Female	1.24	1.08, 1.43	0.0029
	Male	–	–	–
Race/ethnicity	Black	1.14	0.94, 1.38	0.1773
	Hispanic	0.86	0.71, 1.05	0.1328
	Other	1.01	0.61, 1.67	0.9753
	Unknown	1.39	0.95, 2.03	0.0919
	White	–	–	–
SUD treatment	Engaged	5.91	5.05, 6.91	<0.0001
	Not engaged	–	–	–
Diabetes treatment	%Covered days	1.85	1.53, 2.24	<0.0001
Visit	2018	0.28	0.24, 0.33	<0.0001
	2019	0.17	0.15, 0.21	<0.0001
	Baseline	–	–	–

It is important to note that the interaction of engagement and time was significant in the model assessing the relationship between engagement in SUD treatment and HbA1c control. As observed in the Least Squares means results in Table 4, among those engaged and not engaged in SUD treatment, the likelihood of HbA1c control decreases over time. However, the likelihood of control decreases at a faster rate over time in the group which was not engaged in SUD treatment. Furthermore, within each time point, the group engaged in treatment always has a higher likelihood of HbA1c control (at baseline OR = 2.96; 95% CI 2.27–3.85; at 2018 OR = 9.01; 95% CI 7.20–11.27; at 2019 OR = 7.74; 95% CI 6.09–9.84).

**Table 4:** Mean comparisons of measurement period by SUD treatment engagement status 2016–2019 among HCN patients with type 2 diabetes and SUD.

**Engagement status**	**Time point**	**Proportion (%) with HbA1c controlled**	**Odds ratio of HbA1c control as compared to baseline**		
			**Adj. OR**	**95% CI**	***P*** **-value**
Engaged (yes)	2019	10.5	0.28	0.24, 0.34	<0.0001
	2018	16.9	0.49	0.43, 0.56	<0.0001
	Baseline	29.5	–	–	–
Not engaged (no)	2019	1.5	0.11	0.08, 0.15	<0.0001
	2018	2.2	0.16	0.12, 0.22	<0.0001
	Baseline	12.4	–	–	–

## Discussion

Results of this study demonstrate that patients with type 2 diabetes and any SUD were less likely to achieve glycemic control and that engagement in SUD treatment was associated with higher likelihood of diabetes control. These results add to other studies in the literature that documented worse diabetes management, medical complications, and mortality for patients with comorbid diabetes and SUD (13, 20, 38). Results also demonstrate that older age and female gender are associated with higher likelihood of diabetes control (39, 40).

Remarkably, the prevalence of substance use disorder was low in the patient population. Several studies have documented lack of adequate screening of SUD in primary care, causing inadequate identification (21, 41). Some of these practices have been documented as resulting from provider stigma. Additionally, it is possible that even when identified, physicians are less likely to code an SUD diagnosis in the medical records, given the implications for patients (42, 43).

Not surprisingly, patients with type 2 diabetes and SUD that were engaged in SUD treatment were more likely to achieve glycemic control than those not engaged (31). Engagement and retention in substance abuse treatment has been associated with improved adherence to medical treatment, reduced mortality (44) and improved quality of life (45). It is possible that the results seen in this study are driven by adherence to treatment and other behavioral changes described in the literature consequent to SUD treatment. This study did not pursue independent examinations of the effect of pharmacological or behavioral SUD treatment on glycemic control. Data on the impact of psychosocial interventions and pharmacotherapies for SUD in patients with type 2 diabetes are limited (19) and future studies could explore independent effects of type of SUD treatment on glycemic control and explore glycemic control by type of SUD. Results of this study also highlight the decrease of the effect of engagement in treatment in glycemic control over time. It is possible that this decrease is driven by patients moving away or attending a new clinic after the baseline period; patients were categorized as “uncontrolled” due to missing HbA1c values during follow up years.

This work has several strengths. First, to our knowledge this is the first examination of type 2 diabetes and SUD in FQHCs in a Medicaid non-expanded state and provides the chance of further understanding diabetes care quality for patients that are vulnerable to the inequities driven by social determinants of health. Second, results highlight the disparate likelihood of management experienced by patients with diabetes who also have comorbid SUD. Third, findings underscore the importance of SUD treatment and highlight that untreated SUD could affect glycemic control. This is relevant as it can inform treatment resource allocation for SUD within health care centers. Fourth, this study relied and capitalized on the use of EHR and point of care data drawn from FQHCs, offering population level understanding of those most challenged by adverse health outcomes. This low-cost research approach can critically inform systems-level strategies to improve patient outcomes and could lower overall healthcare costs.

The current study has several limitations. First, the design of this study supported findings that are correlational in nature; therefore, causation cannot be concluded. Second, the eligibility timeframe was established by convenience and data availability. The University of Miami, through an agreement with the Miami Clinical and Translational Science Institute and HCN, started receiving data for research purposes in 2016, hence the start date of the eligibility period. Follow up data on HbA1C control were established through 2019, to avoid with confounding effects of COVID-19, starting in 2020. Third, it is possible that additional confounders could explain the associations observed. Diet and exercise, two important factors in glycemic control, are inconsistently documented in the records, and when so, only as unstructured data. This study relied only in a limited data set containing only structured data. Fourth, relying on EHR data is prone to inherent limitations, such as missing data and misclassification. Fifth, while the network of FQHCs represented in this study offers substance abuse treatment in both primary care delivery locations and satellite locations, it is possible that patients received substance abuse treatment outside of the FQHC network therefore precluding those data from being documented in the EHR. Finally, the results of this study might not be generalizable to populations not served in Florida or not served in FQHCs.

In conclusion, this analysis demonstrated that patients with comorbid type 2 diabetes and SUD have worse glycemic control and that untreated SUD could adversely affect diabetes control, thereby shedding light on opportunities to enhance care delivery for patients with diabetes and co-occurring SUD. Understanding diabetes management in the context of comorbidities helps identify opportunities for improving diabetes management outcomes and reducing the risk of diabetic consequences and premature death (46). Revealing the compounding effects of comorbid SUD can aid FQHCs focus their efforts on furthering opportunities for screening of SUDs as well as integrating evidence-based treatments into the mainstream of primary care or delivering brief interventions and linking patients to substance abuse treatment. Additionally, the findings of the current study may have wide-reaching beneficial implications for comorbid diabetes quality-of-care improvement at other FQHC networks across the US. Finally, further research is needed to advance our understanding of the mechanisms by which SUD affects glycemic control and the mechanisms underlying the effects of SUD treatment on diabetes outcomes.

## Data availability statement

The data analyzed in this study is subject to the following licenses/restrictions: electronic health record data analyzed in this study were accessed *via* data use agreement to limit the transfer of identifiable information, and are not available to the public.

## Ethics statement

The studies involving human participants were reviewed and approved by University of Miami Institutional Review Board. Written informed consent for participation was not required for this study in accordance with the national legislation and the institutional requirements.

## Author contributions

VH formulated the research question and drafted the first version of the manuscript. PT routed the data feed *via* University of Miami UHealth IT and provided guidance on datafile and variable management. VH, RS, RD, DP, KC-B, JB, KE, and DF reviewed and decided on operationalization of all study variables. DF provided methodological guidance and approach for the models created for data analyses. RD performed the analyses. RS and RD drafted the methods and results sections. KC-B directs research and DP serves as a data analyst for Health Choice Network, and as such, both provided guidance on understanding data structure and interpreting results. SG and CD conducted literature searches and background review and contributed to the referencing system. All authors provided revisions and approved the submission of the manuscript for publication.
