# Supplementary material for: Untreated substance use disorder affects glycemic control: Results in patients with type 2 diabetes served within a network of community-based healthcare centers in Florida
Source: Front Public Health. 2023 Mar 16;11:1122455. doi: 10.3389/fpubh.2023.1122455 (PMC10060525; doi:10.3389/fpubh.2023.1122455)
Supplement: Supplementary file 1 [file Table_1.DOCX]

**Supplementary Material**

Supplementary Table 1: Criteria for defining population with substance use disorder (SUD)

|  | **Alcohol** | **Chemical** | **Tobacco** |
| --- | --- | --- | --- |
| **ICD-10 Codes** | F10 | F11, F12, F13, F14, F15, F16, F18, F19 | F17 |
| **CPT Codes** | G0443 | J0571, J0572, J0573, J0574, J0575, Q9991, Q9992, S0109 | 99406, 99407, S9075, 4000F, 4001F, 4004F |
| **Medication Key words** | Acamprosate  Antabuse  Contrave  Depade  Disulfiram  Qsymia  Qudexy  ReVia  Topamax  Topiramate  Trokendi | Bunavail  Buprenorphine  Buprenorphine/Naloxone  Methadone  Naltrexone  Sublocade  Suboxone  Subutex  Vivitrol  Zubsolv | Nicotine  Bupropion  Nico Relief  Nico-derm  Nico-relief  Nicoderm  Nicorelief  Nicorette  Nicotine  Nicotinum  Nicotrol  Polacrilex  Varenicline  Wellbutrin  Zonnic  Chantix  Forfivo |

Supplementary Table 2: Medication key words for defining engagement in treatment for type 2 diabetes

| **Metformin** | **Sulfonylureas** | **Glinides/ Meglitinides** | **Thiazolidine- diones** | **DPP-4 inhibitors** | **GLP- 1 receptor agonists** | **SGLT-2**  **Inhibitors** |
| --- | --- | --- | --- | --- | --- | --- |
| Fortamet  Glutmetza  Glucophage  Riomet | Glyburide:  DiaBeta, Glynase  Glipizide:  Glucotrol  Glimepiride:  Amaryl | Repaglinide: Prandin  Nateglinide: Starlix | Rosiglitazone  Avandia  Pioglitazone  Actos | Sitagliptin  Januvia  Saxagliptin  Onglyza  Linagliptin  Tradjenta | Exenatide  Byetta  Bydureon  Liraglutide  Saxenda  Victoza  Semaglutide  Rybelsus  Ozempic | canagliflozin Invokana  dapagliflozin Farxiga  empagliflozin Jardiance |
|  |  |  |  |  |  |  |
|  |  |  |  |  |  |  |
